# Development of a software system for surgical robots based on multimodal image fusion: study protocol

**DOI:** 10.3389/fsurg.2024.1389244

**Published:** 2024-06-06

**Authors:** Shuo Yuan, Ruiyuan Chen, Lei Zang, Aobo Wang, Ning Fan, Peng Du, Yu Xi, Tianyi Wang

**Affiliations:** Department of Orthopedics, Beijing Chaoyang Hospital, Capital Medical University, Beijing, China

**Keywords:** multimodal image fusion, surgical robots, software system, image segmentation algorithm, image registration fusion algorithm

## Abstract

**Background:**

Surgical robots are gaining increasing popularity because of their capability to improve the precision of pedicle screw placement. However, current surgical robots rely on unimodal computed tomography (CT) images as baseline images, limiting their visualization to vertebral bone structures and excluding soft tissue structures such as intervertebral discs and nerves. This inherent limitation significantly restricts the applicability of surgical robots. To address this issue and further enhance the safety and accuracy of robot-assisted pedicle screw placement, this study will develop a software system for surgical robots based on multimodal image fusion. Such a system can extend the application range of surgical robots, such as surgical channel establishment, nerve decompression, and other related operations.

**Methods:**

Initially, imaging data of the patients included in the study are collected. Professional workstations are employed to establish, train, validate, and optimize algorithms for vertebral bone segmentation in CT and magnetic resonance (MR) images, intervertebral disc segmentation in MR images, nerve segmentation in MR images, and registration fusion of CT and MR images. Subsequently, a spine application model containing independent modules for vertebrae, intervertebral discs, and nerves is constructed, and a software system for surgical robots based on multimodal image fusion is designed. Finally, the software system is clinically validated.

**Discussion:**

We will develop a software system based on multimodal image fusion for surgical robots, which can be applied to surgical access establishment, nerve decompression, and other operations not only for robot-assisted nail placement. The development of this software system is important. First, it can improve the accuracy of pedicle screw placement, percutaneous vertebroplasty, percutaneous kyphoplasty, and other surgeries. Second, it can reduce the number of fluoroscopies, shorten the operation time, and reduce surgical complications. In addition, it would be helpful to expand the application range of surgical robots by providing key imaging data for surgical robots to realize surgical channel establishment, nerve decompression, and other operations.

## 1 Introduction

With the accelerating population aging, the number of patients suffering from spinal degenerative diseases, such as lumbar disc herniation and lumbar spinal stenosis, is increasing annually. However, the anatomical structure of the spine is complex and involves critical components such as the spinal cord, nerve roots, and blood vessels. Surgery in this area is unsafe and requires extremely high precision. Rapid advances in surgical robotics can help spine surgeons improve the precision and stability of their surgeries. In recent years, surgical robots employed in spine surgery have been primarily used to assist in pedicle screw placement, such as the TiRobot (TINAVI Medical Technologies Co. Ltd., Beijing, China), Mazor (Mazor Robotics Ltd., Caesarea, Israel), Da Vinci (Intuitive Surgical, Sunnyvale, CA, USA), ROSA (Zimmer Biomet Robotics, Montpellier, France), Excelsius GPS (Globus Medical, Inc., Audubon, PA, USA), and Orthbot (Xin Junte, Shenzhen, China) (1–6). Robotic-assisted lumbar fusion is more precise in pedicle screw placement, has a shorter average operative time, less bleeding, faster postoperative recovery, and reduces radiation injuries to patients and medical staff than the freehand manipulation (7–11).

Currently, spinal surgical robots are mainly used for pedicle screw placement. By importing preoperative CT data and using registration technology to match the actual intraoperative position, surgeons perform the surgery under robotic guidance (3). However, surgical robots rely on unimodal CT images as baseline images, which can only display vertebral bone structure and are incapable of visualizing soft tissue structures such as intervertebral discs and nerves. This has led to a relatively limited scope of application of surgical robots and cannot assist surgeons in the establishment of surgical channels, nerve decompression, and other operations (12). With the continuous development of medical imaging and computer image processing technology, multimodal image fusion has shown significant advantages in the medical field. Multimodal fusion images are valuable for disease diagnosis and preoperative planning (13). In spine surgery, CT/MR image fusion is particularly practical because fused images can accurately provide information about the positions of both bones and soft tissues. However, no studies have reported on preoperative multimodal spine models that can be used for intraoperative registration and navigation of spine surgery robots.

With the rapid development of multimodal image fusion technology and in response to the current limitations in the application scope of spinal surgical robots, this study aims to develop a software system for surgical robots based on multimodal image fusion, which can provide key imaging data for surgical robots to perform operations such as surgical channel establishment and nerve decompression. For spine surgeons and patients, the use of accurate, intuitive, and anatomically rich spine multimodal fusion images to guide surgery can significantly improve surgical efficiency, shorten operation time, and reduce intraoperative complications. Patients will have better postoperative outcomes, reduced medical costs, and significantly improved quality of life.

## 2 Methods

**Figure 1:**
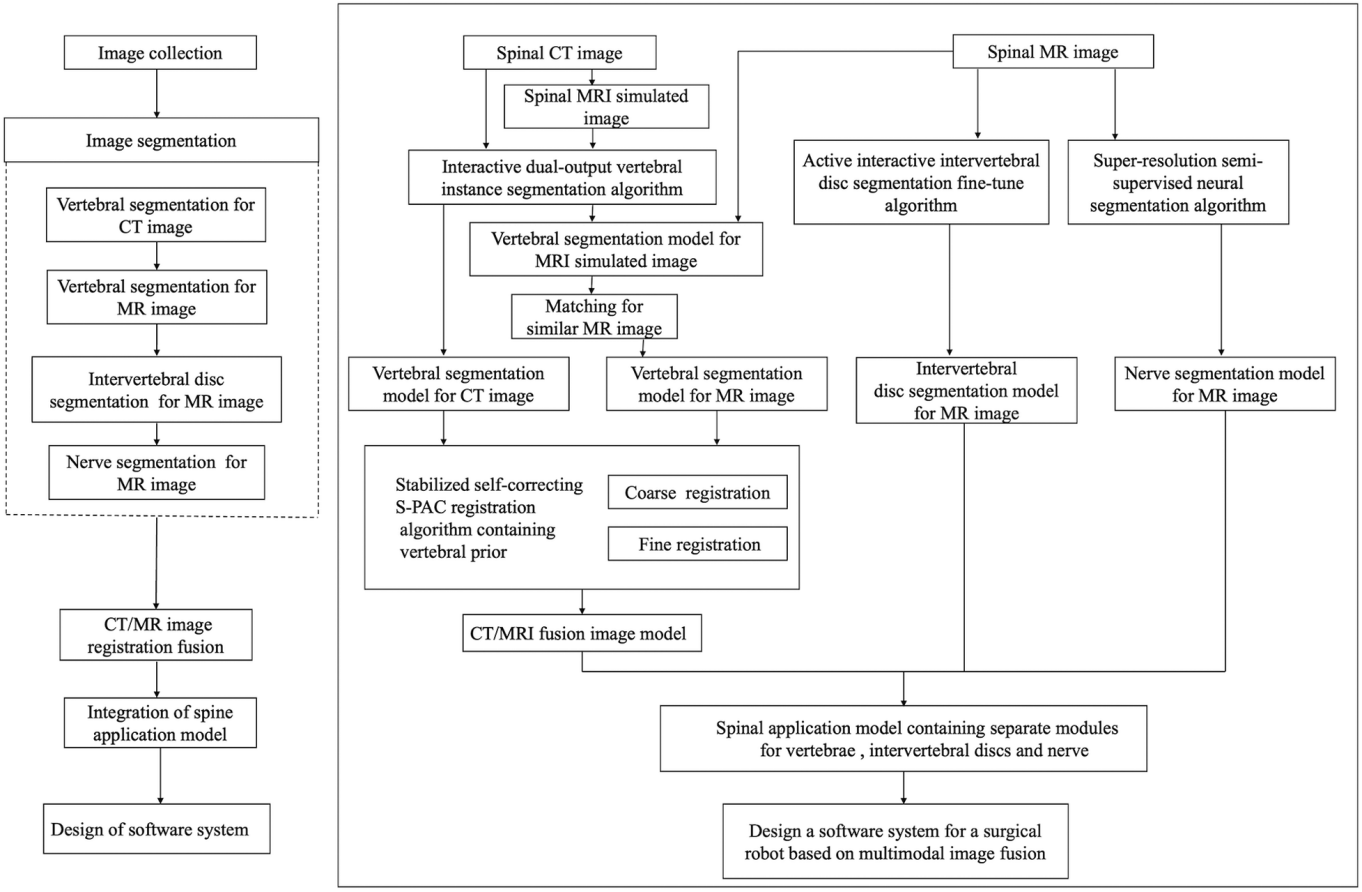
Flow chart showing the development and operation of the system.

The surgical robot software system based on multimodal image fusion consists of six major modules: a data reading and writing management platform, an image visualization platform, a vertebral bone segmentation algorithm platform for CT and MR images, an intervertebral disc segmentation algorithm platform for MR images, a nerve segmentation algorithm platform for MR images, and a CT/MR image registration fusion algorithm platform. The spinal model is constructed using the software system, as follows: input the preoperative spinal CT and MR images of the patient into the software system, and after sequential processing through the four modules of vertebral bone segmentation, intervertebral disc segmentation, nerve segmentation, and CT/MR image registration fusion, construct a spinal model containing independent structures such as vertebrae, intervertebral discs, and nerves. Figure 1 illustrates the development and operation process of the system.

### 2.1 Cases and imaging data

The study will collect preoperative spinal CT and MRI data in DICOM format in 100 patients. The inclusion criteria are as follows: age 20–80 years; male and female sex not limited; clinical and radiological findings consistent with the diagnosis of lumbar disc herniation or lumbar spinal stenosis; informed consent for the study; complete clinical and imaging data. The exclusion criteria are as follows: history of previous spinal surgery; history of spinal deformity; and current diagnosis of spinal infection, tuberculosis, or tumor. These criteria are meticulously applied to ensure the homogeneity and relevance of the patient population involved in our study. The hospital's institutional review board and ethics committee approved this study. Furthermore, all aspects of this study conformed to the principles outlined in the Declaration of Helsinki.

### 2.2 Algorithm construction platform

CT and MRI date in DICOM format are imported into the PYTHON software (version 3.7.0) on the “Chaoyang-Tsinghua Digital and Artificial Intelligence Orthopedic Research Laboratory” (Intel (R) Xeon (R) CPU E5-2620 V4, Titan V 12G GPU, Ubuntu 18.04) for algorithm construction.

#### 2.2.1 Establishment of the vertebral segmentation algorithm for CT images

Our research team has designed an interactive dual-output vertebral instance segmentation algorithm (14). This algorithm uses a self-localization iterative deep neural network approach, leveraging the spatial relationships formed by the spinal chain structure for sequential localization and segmentation of vertebrae. The vertebral segmentation process based on CT images is outlined below: ① Input the CT images of the patient's lesion area, use the initial localization module to determine the localization frame of the first vertebra at the end of the vertebral chain, and complete the segmentation of the first vertebra by the established iterative instance segmentation network. ② Design a self-generating module for the positioning frame that can automatically determine the positioning frame for the next vertebra based on the segmentation result of the first vertebra and then employ the iterative instance segmentation network again to complete the segmentation of vertebrae in this segment. Iterate using the high-precision localization module and the iterative segmentation network to sequentially segment the remaining vertebrae. ③ Finally, use the termination detection module to determine the end of the vertebral chain, stop the iterative segmentation, and complete the instance segmentation of the entire vertebral chain.

#### 2.2.2 Establishment of the vertebral segmentation algorithm for MR images

The overall concept of the vertebral segmentation algorithm for MR images is consistent with that of the iterative vertebral segmentation algorithm for CT images. Notably, improvements have been made to the training process of the dual-output instance segmentation network to enable segmentation and localization on MR images (15). The training process is improved as follows: ① generate MRI simulation images that resemble MRI data using CT image data, ② use vertebral annotations from CT images to train a network model capable of vertebral segmentation on MRI simulation images, and ③ select an MR image sequence with an appearance similar to the generated MRI data and use the trained model to perform precise vertebral extraction directly on the specific MR image sequence, achieving vertebral segmentation in MR images.

#### 2.2.3 Establishment of an intervertebral disc segmentation algorithm for MR images

**Figure 2:**
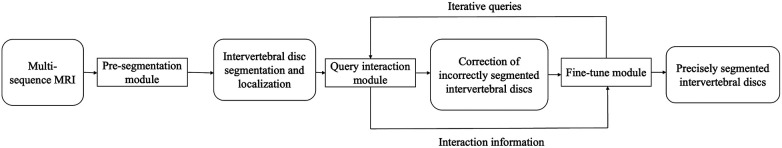
Flowchart of the active interactive intervertebral disc segmentation fine-tune algorithm.

In this study, we will develop an active interactive intervertebral disc segmentation fine-tune algorithm based on deep learning for the segmentation of intervertebral discs in MR images. The flow of this algorithm is shown in Figure 2, which is mainly composed of three parts: pre-segmentation, query interaction, and fine-tune modules. The process of intervertebral disc segmentation based on MR images is as follows: ① The pre-segmentation module initially segments the intervertebral discs and locates their positions from various MR image sequences. ② The location of possible segmentation errors is queried using the query interaction module, and the queried laminae are corrected by clicking using manual interaction. ③ Using this correction click, the pre-segmentation result and the original MR image are fed to the correction network for segmentation result correction and the next query interaction correction until no more laminar slices that require correction can be queried.

#### 2.2.4 Establishment of a nerve segmentation algorithm for MR images

This study employs a super-resolution semi-supervised segmentation algorithm, which is based on the image-processing techniques of the super-resolution generative adversarial network proposed by Ledig et al. (16). We applied this algorithm to nerve segmentation to extract the details of the spinal cord and nerve root edges from MR images. The algorithm can achieve relatively accurate nerve segmentation in high-resolution MRI.

#### 2.2.5 Establishment of the CT/MR image registration fusion algorithm

The current CT/MR image registration fusion algorithms still face challenges in achieving both accuracy and reliability, and stability and practicality require further improvement. To solve these problems, our group has developed a stabilized self-correcting S-PAC registration algorithm containing a vertebra prior. In the pre-experiment, the target registration error (TRE) of this algorithm was measured at 0.91 mm, which met the millimeter-level precision requirements (≤1 mm) for spinal robotic surgery. The registration process consists of the following two steps: ① Establishment of the coarse registration algorithm: based on the surface distance map of the segmented vertebrae, the same segments of vertebrae in the segmented images of CT and MRI vertebrae are matched to avoid segment mismatch. ② Establishment of the fine registration algorithm: in this step, a novel mutual information-based method is designed to match the vertebrae of the same segment after coarse registration for segmentation-based registration. A robust edge-weighted similarity measure was designed for binary rigid registration of individual vertebral to eliminate local minima in the optimization process and improve the stability of the registration algorithm. The vertebral information in the CT image is registered into the MR image as a single vertebra, replacing the vertebral information in the MR images to generate fused images.

### 2.3 Training, validation, and optimization algorithm

Training, validation, and optimization of segmentation algorithms: ① Using manually segmented results by spine surgeons as the gold standard, the segmentation algorithms are quantitatively evaluated through tenfold cross-validation. All vertebrae were randomly divided into 10 groups, rotating through 10 cycles of training and testing, with each cycle reserving one group as the test set. The remaining nine groups are split into training and validation sets in an 8:2 ratio. ② Evaluation of the test results. The test results of vertebral segmentation algorithms for CT and MR images and nerve segmentation algorithms for MR images were quantitatively evaluated in terms of accuracy using the Dice coefficient and average surface distance metrics. Four quantitative evaluation metrics are used for the test results of intervertebral disc segmentation algorithms for MR images, which include the values of Dice coefficients after querying and correcting for the *n* layers (In- Dice), Hausdorff distance (HD) 95% value after querying and correcting *n* layers (In-HD95%), Dice coefficient value after a single 3D correction (SC-Dice), and HD95% value after a single 3D correction (SC-HD95%). ③ Finally, spine surgeons assess the accuracy and practicality of the segmentation results. Twenty-five vertebrae will be randomly selected from the vertebral labeling results of the training data as the gold standard and mixed with the segmentation results. Spine surgeons are unaware of the gold standard and the results of network segmentation during the evaluation process. The identified differences between the two sets were analyzed, providing feedback for further optimization of the registration algorithm.

The sheep spine specimen is used for the validation and optimization of the registration fusion algorithm for CT and MR images. The process is as follows: (I) The sheep spine specimen is fixed in a plastic container that is not visible in both CT and MR images. Cod liver oil, which can be visualized by CT and MRI, is selected as the reference marker, and fixed on the transverse and spinous processes of the vertebrae. (II) CT is performed on the specimen, and the body position is divided into the supine group (y) and prone group (f). The spinal posture is divided into the upright group (1) and scoliosis group (2), and three scans (1y, 2y, and 1f) are performed. The scanning layer thickness is changed by post reconstruction, and the layer thickness is categorized into 0.6 (a), 1.2 (b), and 2.4 mm (c) groups, and further combined with the above scanning results into five groups (a1y, a2y, a1f, b1y, and c1y). The CT scan time is counted from the beginning of the CT scan to its completion. (III) High-resolution MRI is performed on the specimens, and the MRI scanning time is also counted. The scanned layer thickness and layer spacing are changed by post reconstruction, and the layer thickness is categorized into two groups of 0.8 mm (a) and 2.0 mm (b). The scanned layer spacing is categorized into 1 (no layer spacing) and 2 (layer spacing = 50% layer thickness), further combined with the above-mentioned scanning results, and categorized into three groups (a1, b1, and a2). (IV) The results of the registration with the benchmark markers are considered the gold standard, and the algorithms of this study are then evaluated. ① Accuracy evaluation: TRE and fiducial registration error (FRE) of the a1y group are calculated using the benchmark marker registration results as the gold standard. Using a blinded approach, spinal surgeons assessed the registration outcomes of both the gold standard algorithm and the proposed algorithm through a survey questionnaire. ② Stability evaluation: TRE = 2 mm is used as the threshold for successful registration, and the registration is repeated 100 times for the a1y group to calculate the success rate (%) of the registration. ③ Reliability evaluation: using the benchmark marker registration results as the gold standard, calculate the TRE and FRE of the fusion images of each group and then horizontally compare the TRE and FRE between the groups. ④ Practicality evaluation: analyze whether data acquisition, registration time, and image quality can meet clinical needs. The evaluation results are analyzed and feed back to further optimize the registration algorithm. ⑤ Finally, spine surgeons evaluate the accuracy and practicality of the fused images.

### 2.4 Integration of the spine model and software design

On the “Chaoyang-Tsinghua Digitalization and Artificial Intelligence Orthopedic Research Lab” specialized workstation [Intel(R) Xeon(R) CPU E5-2620 V4, Titan V 12G GPU, Ubuntu 18.04 operating system], a software system is developed using PyQt5 5.15.4, SimpleITK 1.2.4, and VTK 9.2.2. This system integrates fused image models with corresponding vertebrae, intervertebral discs, and nerve segmentation models. Subsequently, a comprehensive software platform for spine modeling is established, comprising a data reading and writing management platform, an image visualization platform, a vertebral bone segmentation algorithm platform for CT and MR images, an intervertebral disc segmentation algorithm platform for MR images, a nerve segmentation algorithm platform for MR images, and a CT/MR image registration fusion algorithm platform.

An additional 20 patients who meet the criteria will be included, and the corresponding spine models will be constructed and validated using the spine model integration software platform. Spine surgeons will evaluate the accuracy and practicality of the spine models. The spinal surgeons will manually segment vertebrae, intervertebral discs, and nerve structures. Using anatomical landmark points, they will perform CT/MR image registration and fusion to construct manually segmented spine models, which will serve as the gold standard for spine models. The gold standard will be mixed into the spine models constructed using the software system. During the evaluation process, they will not be informed about which ones are the gold standard and which ones are the results of network segmentation, ultimately comparing the differences between the two groups.

## 3 Discussion

This study aims to establish and optimize image segmentation and registration fusion algorithms and subsequently develop an integrated software system that includes vertebral segmentation algorithms for CT and MR images, intervertebral disc segmentation algorithms for MR images, nerve segmentation algorithms for MR images, and CT/MR image registration fusion algorithms. This integrated software system will be used to construct a multimodal spine model containing independent modules of vertebral bones, intervertebral discs, and nerves and then conduct clinical application research. Developing a software system for surgical robots based on multimodal image fusion is important. The clinical application of this software system will provide key imaging data for surgical robots to perform nerve decompression and other operations, which will further improve surgical accuracy and reduce surgical complications. Our group has made some research progress in the preoperative planning system, navigation system, and vertebral shaping system, which is a critical step toward the successful application of this software system in spinal surgery.

Pedicle screw placement is a critical step in spinal internal fixation surgery because misplaced screws may lead to neurological deficits or vascular injury (17, 18). The reported rates of screw misplacement vary significantly across different studies. Castro et al. (19) reported a misplacement rate as high as 40%. The complication rate due to misplaced screws ranges from 0% to 54% (20–23). Many studies have confirmed that robot-assisted pedicle screw placement has significant advantages over traditional fluoroscopic techniques, with an accuracy rate ranging from 93% to 100% (10, 11, 24–26). Kantelhardt et al. (27) first reported an accurate placement rate of 94.5% for robot-guided pedicle screw placement compared with a 91.4% accuracy rate for traditional screw placement. Several subsequent studies have also demonstrated that robot-assisted screw placement is superior in accuracy to traditional methods and results in less tissue damage during surgery, thus providing a better prognosis for patients (28–32). Moreover, undoubtedly, the future of spinal robotics should not be limited to assisting surgeons in pedicle screw placement alone. They should also assist in performing different surgeries for various spinal disorders. Wang et al. (33) and Lin et al. (34) reported that robot-assisted percutaneous kyphoplasty has advantages such as higher puncture accuracy, shorter channel establishment time, reduced radiation exposure, and lower bone cement leakage. Currently, percutaneous endoscopic discectomy (PTED) is a commonly used minimally invasive surgery for the treatment of lumbar disc herniation, and the difficulty of this surgery lies in the establishment of the surgical channel, which requires multiple fluoroscopy and punctures to reach the target position (35). Yang et al. (36) found that robot-assisted guided PTED, compared with conventional c-arm fluoroscopy-guided PTED, had fewer punctures (1.20 ± 0.42 vs. 4.84 ± 1.94), fewer fluoroscopy (10.49 ± 2.16 vs. 17.41 ± 3.23), and shorter surgery time (60.69 ± 5.63 vs. 71.19 ± 5.11 min). Importantly, our surgical robot software system, through multimodal image fusion technology, achieves three-dimensional visualization of the surgical area's anatomical structures, providing better guidance for surgery and maximizing the safety of the surgery.

Currently, the localization and navigation functions of spinal surgery robots are mainly based on CT images. CT images provide excellent visualization of bone structures but post challenges in distinguishing soft tissues such as nerves and ligaments. In contrast, MR images offer good visualization of soft tissues. Owing to the lack of local anatomical information, the application scope of robots in spinal surgery is extremely limited (12). However, some researchers have attempted to address this issue using CT/MRI fusion images. Their research results indicate that fusion images can better present the relationship between the bony and soft tissue components of lesions (37–39). This can serve as a valuable tool to enhance the accuracy of surgical planning.

The construction and application of spine models based on CT/MR image fusion technology involves several key technologies, such as vertebral bone segmentation, intervertebral disc segmentation, nerve segmentation, and multimodal image registration and fusion. ① Vertebral bone segmentation. Previous studies using traditional CT vertebral segmentation techniques have achieved precise segmentation of simple vertebral regions. However, challenges remain in segmenting complex areas such as tightly connected facet joints. Although some progress has been made in deep learning-based vertebral segmentation technology, it also suffers from the problems of long consumption time and poor accuracy (40–42). To address this issue, our research team designs an interactive dual-output vertebral instance segmentation algorithm. The results of algorithm training and optimization show relatively high overall and local segmentation accuracy. Compared with existing methods, the training speed of the algorithm is improved by 2.7 times, and the segmentation speed is improved by 4 times (14). ② Intervertebral disc segmentation. Current research on intervertebral disc segmentation technology requires further investigation. Recently, a research team developed a fully automated intervertebral disc recognition and segmentation algorithm based on iterative neural networks (43–46). However, local accuracy within the surgical area, which is crucial for spinal robot surgeries, remains unclear. To address this problem, our group designed an active interactive multimodal intervertebral disc fine-tune algorithm, which achieved an I9-Dice coefficient of 92.50% ± 1.82% for high-resolution intervertebral disc data in the pre-experiment. ③ Nerve segmentation. In addition, fewer studies have focused on nerve segmentation. Existing segmentation algorithms struggle to achieve complete and high-precision segmentation of neural tissues in routine clinical images. Although intraoperative neural automatic extraction algorithms have been designed, they are not suitable for preoperative planning (47). This study applies a super-resolution semi-supervised segmentation algorithm to nerve segmentation to achieve more precise nerve segmentation. ④ Multimodal image registration and fusion. Many researchers have explored the possibility of spinal CT/MR image registration fusion to integrate the anatomical information of bone and soft tissues. However, the results from these studies have not been ideal. Challenges include poor accuracy, reliance on manual vertebral joint matching, and overall long registration times (48, 49). Some scholars have applied the MIND Demons algorithm to deformably register soft tissues from MRI and fuse them into CT images; however, its similarity measure is unstable in multimodality at the time of registration (50). Our group designs a self-correcting S-PAC registration algorithm with vertebrae prior, which may help solve the current CT/MR image registration algorithm, in which combining accuracy, stability, reliability, and practicality is difficult. In this study, we used sheep spine specimen to validate and optimize the registration fusion algorithm for CT and MR images. Although the sheep spine specimen is a little different from the human body structure, it can meet our current study requirements. Certainly, we will further optimize and validate the algorithm on human cadavers in the future when conditions permit.

The current study protocol aims to preliminarily develop a software system based on multimodal image fusion, but its practical application to spinal surgery robotics still needs to overcome many challenges. Firstly, we only included patients who met the diagnosis of lumbar disc herniation or spinal stenosis, which was aimed at guaranteeing the validity and stability of the algorithm lightweight. Thereafter, we will further incorporate various types of patients such as spinal deformity, history of spinal surgery, spinal fracture, spinal infection, and spinal tumour, to continuously improve the clinical applicability of the system. Furthermore, contemporary deep neural network models commonly employed are characterized by large size and parameter count, requiring long computing time and high GPU computing power, which limits the practical application. In recent years, lightweight of algorithms has become an important optimization direction. He et al. (51) constructed a lightweight algorithm by reducing the parameters related to the algorithm, which has the advantages of fewer parameters, smaller size and faster training speed. In the future, in addition to upgrading our equipment and network on time, we plan to use lightweight algorithms to make our system faster and more clinically applicable. Finally, it's noteworthy that currently, no automated or semi-automated spinal surgery robot capable of soft tissue manipulation has been developed. Consequently, in the short term, the system will primarily provide navigation functions for the surgeon's manual operation. We believe that this system will play a greater role in the future with the further development of robotics.

Until now, spinal surgical robots have commonly utilized single-modal spinal CT images as intraoperative baseline images. No studies have reported on multimodal, multi-independent module spine models that can be employed for intraoperative registration and navigation of spinal surgery robots. This study faces numerous challenges that must be overcome, with many issues urgently requiring resolution. Nevertheless, this study has vast prospects for application. The application of the outcomes of this study to spinal surgical robots can significantly expand the scope of their use. It can facilitate robot-assisted operations in spinal surgery, such as the establishment of surgical channels and neural decompression. This would notably enhance the precision, effectiveness, and minimally invasive nature of intraoperative surgeries. This makes spine surgery robots more clinically applicable, ultimately benefiting a larger population of patients.
